# Spatial dynamics in the classroom: Does seating choice matter?

**DOI:** 10.1371/journal.pone.0226953

**Published:** 2019-12-31

**Authors:** Jason S. Bergtold, Elizabeth A. Yeager, Terry W. Griffin

**Affiliations:** Department of Agricultural Economics, Kansas State University, Manhattan, KS, United States of America; Universitá degli Studi di Bergamo, ITALY

## Abstract

This study examines peer and seating effects on overall class performance and exams from a spatial perspective for principles of economics courses at a major Land Grand institution in the Midwest. Both spatial and student specific factors were identified that impact performance. The spatial relationships in the classroom were explored to determine if students’ peers and seating choice affect their performance. Endogenous spatial peer effects were only found to impact performance on the first exam. Other findings found gender, being an economics major, sitting in the back of the class, and the year the class was taken all impacted overall exam and class performance.

## Introduction

A number of studies have examined factors affecting students’ performance in principles of economics classes [1–7]. Previous factors of interest have included: academic factors (such as performance on exams, performance on homework, attendance, use of outside assistance, class status, major, special accommodations, GPA, SAT/ACT test scores, instructor, and transfer credits); demographic factors (such as ethnicity, age, gender, financial status, being a veteran, and permanent place of residence); and spatial factors (such as proximity to the front of the classroom, who you sit by, and performance of students around you). Despite the popularity of this topic, results are still somewhat inconclusive in regards to the significance and effect of academic, demographic, and spatial factors in the classroom, as well as controlling for the same instructor and teaching methods, which may vary over time and institutional environment.

This study specifically contributes to previous literature through the investigation of spatial effects through peers and seating preference on class performance in a principles of microeconomics class. To the authors’ knowledge, despite interest in seating location, proximity to the front of the classroom, and who you sit by, no studies have specifically looked at the different impact each student neighbor may have on academic performance. Even outside the academic literature, most spatial models assume that spatial effects are isotropic, or direction does not matter, whereas anisotropic models allow for directionality to be captured [8]. In stadium style seating, it is possible that a student might be able to see exams or other coursework of classmates sitting near them, leading to academic dishonesty issues. More than likely, students who are allowed to choose where they sit may have direct social relationships through fraternal organizations, clubs, majors etc. to students sitting in close proximity, leading to peer effects, as these students may study together, share notes, or distract each other in class.

The overall purpose of the current study is to contribute to the previous literature by examining explicit spatial factors, such as peer effects and seating choice in the classroom affecting student performance in principles of agricultural economics courses at a major Midwestern Land Grant institution. Objectives include: (i) identify factors impacting performance on each of four exams (three regular exams and a comprehensive final exam); (ii) identify factors impacting overall performance in the classroom; and (iii) explore anisotropic spatial relationships in the classroom to identify if peer effects or spatial arrangement impacts student performance.

## Previous literature

Student success in introductory or principles of economics courses have intrigued researchers for decades. [1] analyzed the performance of students across five sections of a principles of economics course to identify whether the course should be presented the same way to economic majors versus non-majors. They considered five factors expected to affect grades in the course: number of credit hours the student had successfully completed (interpreted in the current study as class status); student’s major; grade point average (GPA); instructor; and gender. The results of their study found that a student’s GPA was the most significant variable to predict the final grade.

Peer effects or peer influences is a large body of research that encompasses student achievement as well. [9] and [10] examine peer effects in the academic performance of students who are randomly assigned roommates at Williams College (a highly selective school) and Berea College (with a very heterogeneous population), respectively. Their results were mixed. [9] found that students who were in the middle of the SAT score distribution performed worse if their roommate was in the bottom 15% of the verbal SAT score distribution and that verbal SAT scores were more closely linked to peer effects than math SAT scores, but they did not provide a justification for this. [10] found some evidence that grades, study efforts, and beliefs regarding the importance of education may be influenced in the short run by peers. This is reasonable given peer effects can represent not only students mentoring or teaching one another, but through knowledge spillovers in a dormitory room or classroom that can influence how an instructor may react to certain students or areas of the classroom [11]. There are multiple channels in which peer effects can be distributed.

Researchers have also examined attendance and related policies on academic performance in economics courses. [6] looked at the impact of mandatory attendance policies on grades, as well as the impact of absentee rates on exam scores across four principles of microeconomics courses. Additional variables of interest were class size, major, gender, cumulative hours completed, taking a prior economics course, number of courses withdrawn from, transfer student status, SAT scores, GPA, exam scores throughout the semester, class average, and absences before each exam. Exam scores, GPA, and SAT scores were significant predictors of performance while attendance and absentee rates did not impact grades.

In addition to the more general academic and demographic variables of interest, a new wave of research has focused on the spatial aspects of the classroom or where students are sitting. [5] found that students who indicate a preference for sitting in the front of the classroom have a higher probability of receiving As and those who have a preference for sitting in the back of the classroom have a higher probability of receiving Ds and Fs, regardless of whether they actually sat in the back of the room or not. [7] examined the relationship between seating location within the classroom (front, middle, or back), class preparedness (reviews text before test, reviews notes before class, and math level), outside assistance, gender, and class standing for an introductory agricultural economics course. They found that students who sat in the front or middle of the room performed better, as did females, students who reviewed the text before tests and those students who had completed higher level math courses. Surprisingly, their results indicated that students who sought outside assistance performed worse; however, this may be due to these students struggling with the material and recognizing early on they needed extra assistance.

[12] considered grades and attendance across five different seating configurations: row, column, front of the room versus back of the room, center of the room versus the perimeter of the room, and middle of the room versus sides of the room. Their findings indicated that students who sat in the middle or central part of the room had better grades and attendance and that female students generally had better attendance, although females did not necessarily perform better academically. [12] acknowledge that future research to identify why students choose specific seats or locations in the classroom would be useful.

[13] take a different approach in that their focus is on Brazilian public elementary schools. Despite a different sample of students, the research results support what is often observed in college classrooms across the U.S. The researchers found that students sitting in the front of the room performed better academically and had fewer absences. The students were questioned regarding their reason to choose a seat in a particular location. Motivation for learning was identified as the primary reason for choosing a seat in the front of the room. Friendship was the primary reason students chose to sit in the middle of the classroom, and social isolation was identified as the primary reason students chose to sit in the back of the classroom.

[14] examined different factors influencing test performance in large psychology classes in a university setting. They found that seating location had no significant impact on grades. They found that the difficulty of the exam, the timing of test, expected grade and amount of time studied were significantly correlated with test grades in the class.

[15] examined student seating location on student engagement, attention, classroom learning experience and course performance in a large lecture hall university setting. Seating location was identified as front of the room, middle of the room and back of the room. The researchers found that seating location had an indirect effect on classroom performance through paying attention in class, learning orientation, classroom self-esteem and intrinsic motivation for attending class. Seating location only had a direct and positive significant effect for students sitting in the front of the classroom.

[16] examined the effects peers in the classroom may have on student performance in a university setting in South Korea. Students in this setting, as in this study, were able to choose where they sat in the classroom. The authors correct for this endogenous selection in a model that captures both observed and unobserved peer effects. They explicitly take account of spatial isotropic peer effects of the students sitting around a particular student, but find the average performance of these students has no significant impact on students’ performance in the class. On the other hand, they do find a positive and statistically significant effect of peer exam performance for peers that sit next to them in class (an anisotropic effect).

[17] examined the proximity effect or how close you sit to a lecturer for a set of six economics courses of mid to small size in a community college setting. They found that the row you sat in had no statistically significant impact on academic performance in these classes. This is in contrast to results found by [5] who found that there a proximity effect was significant for those sitting in the front of the classroom for a large lecture hall type of class. [17] conclude that proximity effects may exist in large classrooms, but may not show up in small or medium size classes.

This study attempts to contribute to the literature on seating arrangement and peer effects by identifying factors impacting performance on exams and overall performance in a large principles of microeconomics course. Furthermore, we explore the use of isotropic and anisotropic spatial relationships in the classroom to identify if students’ peers and seating choice affect their performance. Secondary classroom data from a course in which the material has been taught in a similar fashion for the past few years is used, helping to control for differences in teaching methods.

## Data

This study utilizes secondary data regarding gender, grades, level of math completed, GPA, class rank, major, and seating arrangement obtained from a principles of agricultural economics and agribusiness course taught by the same professor from a major Land Grant university in the Midwestern United States during the Fall 2009 and 2010 semesters [18]. The data were collected from course performance (grades) and seating charts (seating arrangement); and was supplemented with data from the university’s student information system to control for other factors (gender, level of math completed, GPA, class rank, and major). Each semester, at least 160 students were enrolled in the course. In addition, the professor that taught the course has over 30 years of teaching experience and is recognized as a distinguished teaching scholar by the university, as well as other national organizations. The Committee on Research Involving Human Subjects / Institutional Review Board (IRB) for Kansas State University found the study to be exempt under federal regulation 45 CFR 46.101, paragraph b, category 4. The application number was 8268. The Office of the Registrar waived the requirement for informed consent and all data were anonymized prior to analysis. A minimal data set, S1 Data, is provided online as a supplement.

Summary statistics are reported in Table 1. Data were available for 161 and 186 students in 2009 and 2010, respectively. The percentage of female students was slightly higher in 2009 compared to 2010, 47.83% and 42.47%, respectively. The number of students who were recorded as an agricultural economics major was greater in 2010 as was the number of students not in the College of Agriculture. The course was comprised of a higher percentage of freshman in 2010 compared to 2009.

**Table 1:** Summary statistics for principles of agricultural economics.

Demographics	2009^a^	2010^a^
Female	47.83%	42.47%
	(50.11%)	(49.56%)
Current GPA	2.73	2.79
	(0.89)	(0.82)
Algebra	34.16%	37.63%
	(47.57%)	(48.58%)
Ag Econ Major	32.30%	19.89%
	(46.91%)	(40.03%)
Non Ag Major	10.56%	5.91%
	(30.83%)	(23.65%)
Freshman	45.96%	51.08%
	(49.99%)	(50.12%)
Sophomore	32.30%	29.57%
	(46.91%)	(45.76%)
Junior	15.53%	12.90%
	(36.33%)	(33.61%)
Senior	6.21%	6.45%
	(24.21%)	(24.63%)
Homework 1	81.57	76.60
	(16.72)	(15.55)
Exam 1	71.50	70.98
	(14.59)	(12.09)
Homework 2	70.90	77.02
	(18.25)	(13.67)
Exam 2	69.75	66.53
	(17.15)	(15.24)
Homework 3	76.47	68.27
	(18.6)	(17.77)
Exam 3	75.24	72.71
	(16.94)	(16.32)
Homework 4	82.70	82.10
	(24.17)	(18.57)
Comprehensive Final Exam (Exam 4)	78.93	74.12
	(14.57)	(12.26)
Overall Homework Grade	77.91	76.00
	(15.58)	(11.79)
Final Course Grade	78.11	76.05
	(13.28)	(11.79)

^a^ The reported statistics represent the sample mean and standard deviation (in parentheses).

Over the course of each semester, the students took three in-class exams (exams 1–3) and a comprehensive final examination (exam 4). Course grades were also comprised of attendance, homework assignments, and quizzes before each exam. For this study, the quiz and homework grades were combined in the analysis. The authors have no reason to believe this will impact the results. All quiz and homework grades were graded on a 10 point scale. The attendance score is intuitively interesting in general; however, this portion of the dataset had a number of inconsistencies and was not readily usable [18].

Class was held in a traditional, lecture style classroom with individual desks. The room seats 201 students and is equipped with a computer for the instructor, projector, document camera, and whiteboard. Students were able to choose their seat at the beginning of the semester, but were asked to sit in that seat for the remainder of the semester. Consistency in seating assignment was maintained by attendance grades that were based on the seating chart. Because the students were allowed to choose where they sat, it was not a random assignment; however, with approximately 50% of students enrolling as freshman it is plausible that the majority of students did not know each other prior to enrolling in this course. In a truly random seating experiment, there is the potential loss of peer effects that may arise from social relationships or people who would work together. A limitation of this study and an area for future research is that information was not collected on how well the students knew their peers in the class nor if they studied or interacted with them in other ways outside of the classroom. As [19] detailed, there may be a reflection problem that makes it difficult to separate out the selection effect from an actual peer effect if this is the case.

The classroom has a total of 15 rows of seating with up to 14 seats per row. When examining grade distribution across space, grades varied considerably across the classroom giving evidence of some random spatial variation in the grades. The classroom has a slight incline from the front to the back such that each row of seating from the front to the back of the classroom is slightly higher than the row before it (i.e. stadium style seating). In addition, seats are in perfect columns within the rows, lining up from the front to the back of the classroom. That is, the seats are not offset from each other from row to row.

## Methods

To assess the factors impacting performance on exam grades and overall class performance, a model of student performance that is designed to capture student specific, performance, and spatial factors is presented here.

### Performance model

A student’s performance, as demonstrated in the literature review, is dependent on a number of explanatory factors. These include student specific demographic factors (***X***_*i*_, e.g. gender, mathematical preparedness, student rank, college and major); student specific class performance factors (***Z***_*i*_, e.g. homework grade and GPA); spatial heterogeneity (***R***_*i*_, e.g. seat location), and spatial peer effects. Let a student *i*'s performance on a specific exam or final course grade be given by *G*_*i*_. Then, assume that *G*_*i*_ is related to these explanatory factors via the following functional relationship:

$$G_{i}=\alpha +\rho E(G_{i}|A_{i})+\beta \prime X_{i}+\gamma \prime Z_{i}+\theta \prime R_{i}+u_{i},$$ (1)

where (***α***, ρ, ***β***, ***γ***, ***θ***) are parameters to be estimated and *u*_*i*_ is a mean zero IID error term capturing the unmodeled portion of a student’s performance on an exam or class. Following [19], *E*(*G_i_*|***A***_*i*_) is an endogenous peer effect that is affected by characteristics ***A***_*i*_ of the reference (i.e. social or peer) group a person is associated with, where ***A***_*i*_ is a vector of characteristics describing the reference group(s). This effect examines how “the propensity of an individual to behave in some way varies with the behavior of the group” ([19], p. 532). In this study, the endogenous effects capture the impact on performance from the group of students that is clustered around a particular student in the classroom. That is, the peer effect in our study examines the effect of peer performance on examinations and in the overall class on an individual student’s performance on exams and in the overall class. As stated by [19], Eq (1) gives rise to a pure endogenous effects model. To be able to identify the marginal impact, ρ, of the endogenous effect, [19] states that the individual specific variables (***X***_*i*_, ***Z***_*i*_) must not be functions of ***A***_*i*_ and the conditional means of (***X***_*i*_, ***Z***_*i*_) must not be linearly related or vary with ***A***_*i*_ (almost everywhere). As mentioned earlier, we were not able to collect data on potential reference groups. Thus, ***A***_*i*_ is unobserved in our sample. We are not able to directly identify the impact of reference groups (i.e. exogenous effects) or their characteristics on students’ class performance. To deal with this issue, we adopt a nonparametric approach to model the endogenous peer effect and test for potential unobserved effects from reference groups. The identification challenges are further discussed below.

Another spatial factor of particular interest in this paper is the seat row or proximity effect, a factor capturing spatial heterogeneity that has been examined in prior studies. There are 15 rows of seating in the classroom. Given the limited degrees of freedom and the number of explanatory factors, the classroom was broken into three sections: front (rows 1 to 5), middle (rows 6 to 10), and back (rows 11 to 15). Binary variables are included in the regression given by Eq (1) for the middle and back sets of rows in ***R****i* to model “row” effects. The sign on these variables is expected to be negative and greater the further back a student sits in the classroom [7,12,13]. While the “row” effect is of interest, unique to this study is the effect of who a student sits by or potential endogenous peer effects.

Finally, it should be mentioned that the difficulty level of exams may change as the semester progresses. For example, the final, which was comprehensive, may be more difficult than the three midterm exams, due to the extent of material covered. To be able to take account of the differences in exam performance, separate regression models were estimated for each exam and one for the overall course grade.

### Isotropic versus anisotropic spatial peer effects

Most spatial models assume that spatial effects are isotropic, meaning that direction does not matter [8]. While this may be true in certain situations, it is the hypothesis here that who a student sits by matters. If spatial effects are significant, this implies that not only where a student sits, but who sits around that student will impact their performance. That is, there exists an endogenous peer effect. For example, a student who sits by friends or colleagues (to the left or right of them) may improve their performance, given that these individuals may study and do homework together, having a positive spillover effect for the *i*^th^ student. That is, the better performance of neighboring students and their understanding of the material, may provide better performance and understanding for the *i*^th^ student. This was the type of peer effects examined by [16]. Other types of peer effects may exist, as well. Recognizing that the chosen seat assignment is not random makes the seating choice and corresponding peer effects endogenous, and the endogeneity of the spatial effects must be modeled appropriately.

Given the stadium style seating in the classroom (i.e. upward incline from the front to back of the classroom), it may be the case that student performance may improve due to academic dishonesty, as well. A student in the classroom, if positioned correctly, could potentially see an exam by a student in the row below them (or beside them). Thus, the *i*^th^ student may perform well on an exam by copying another student’s exam in the row below (or the seat next to them). Thus, the nearest neighbors for the *i*^th^ student can impact their performance and each neighbor may have a different impact. That is to say, the spatial effects of neighbors may be anisotropic. To examine if there is a differential effect of each neighbor, we examine two models of spatial peer effects: an anisotropic and isotropic model. In the isotropic model, the spatial effects in all directions are assumed to be the same. That is, the spatial effect is directionless.

To model the anisotropic spatial effects of the *i*^th^ student’s neighbors, we use spatial lags of attainment or student performance (*G*_*i*_). Thus, the peer effect examines the spillover of the performance of a student’s performance on the students around them. We were not able to collect data on if students studied together, belonged to the same social organizations, social networks, or direct interactions among students in the class in the data for this study. Thus, the peer effects measured are only able to capture potential spillover effects of student performance on others seated around them. The latter relationships and peer effects are areas for future study. It is assumed that only the immediate neighbors in the seats to the *i*^th^ student’s right (R) and left (L), as well as the seats in the row below to the (diagonally) right (DR) (i.e. in front of the student one row and to the right), front (F), and (diagonally) left (DL) (i.e. in front of the student one row and to the left) of the person impact their performance. The effects of students sitting behind a student are not considered in this study. To model these potential directional peer effects spatially, five separate directional weight matrices are developed following [8] that indicate the neighbors for a given seat in the classroom. These include weight matrices for seats to the *i*^th^ student’s right (**W**_R_), left (**W**_L_), diagonally right and in front of (**W**_DR_), front (**W**_F_), and diagonally left and in front of (**W**_DL_). All weight matrices are row standardized as is convention in the spatial econometrics literature for interpretation purposes [8,20].

To incorporate the spatial effects into the performance model, spatial lags for the five spatial directions being modeled are incorporated in Eq (1), by letting:

$$\rho E(G_{i}|A_{i})=\rho_{R}W_{R}G_{i}+\rho_{L}W_{L}G_{i}+\rho_{DR}W_{DR}G_{i}+\rho_{F}W_{F}G_{i}+\rho_{DL}W_{DL}G_{i}$$ (2)

where ρ_*j*_ for *j* = R,L,DR,F,DL are spatial dependence parameters representing the directional spatial peer effects among students in different directions [8,19]. These can be interpreted as nonparametric estimates of endogenous peer effects of student performance following [19]. [19] notes that this type of pure endogenous effects model makes sense in studies with large group interactions, which can include large principles of economics classes if the likelihood of taking the class for a given student is independent (or relatively so) of another student’s choice [19]. This is likely the case in our study, as many of the students are new freshman, as well as transfer students (who are likely sophomores or juniors) who are taking the class there first semester at the university and who registered for the class prior to arriving at the university.

To examine if the endogenous peer effects are directional, the anisotropic model can be compared to an isotropic model. In the isotropic model:

$$\rho E(G_{i}|A_{i})=\rho WG_{i},$$ (3)

where ρ is the endogenous effect parameter and **W** is a spatial contiguity matrix that identifies spatial neighbors to the immediate right, left, diagonal right in front, front, and diagonal left in front. As with the other weight matrices, **W** is row standardized [8]. In an isotropic model, it is assumed that the endogenous peer effect is the same in all directions away from the student (e.g. the spillover effects from beneficial study habits or interactions is the same from all neighbors).

### Identification and estimation

Identification of the endogenous effect is important in order to make a claim that peer effects exist in the classroom. The conditions for identification of the pure endogenous effect were discussed earlier. Here we make a case for identification. As stated in the data section, most of the explanatory variables are individual specific, which is particularly true for ***X***_*i*_, which are primarily student demographics. Gender may be seen as a reference group, but the likelihood of a student being male or female was roughly the same in the class each year. Elements of ***Z***_**i**_, including performance on homework and cumulative GPA are reported as individual specific. The explanatory variables in ***Z***_*i*_ (and some in *X*_*i*_) may be impacted by reference groups, such as membership in fraternal organizations or clubs; but, as stated earlier, the principles level class examined here is taught in the Fall semester and is a first semester course for many freshman and transfer students, which usually represents 70+ percent of enrollment in the course. Thus, many of the students register for the class prior to attending the university fulltime This does not necessarily rule out the existence of potential selection bias due to students sitting together due to the presence of reference groups (e.g. fraternal organizations). If selection bias did exist in the model, we would expect the mean of the peer effect to shift based on the reference group as discussed earlier. To test for this, we estimated both the isotropic and anisotropic models in the paper assuming the peer effect was random, i.e. ρ was treated as a random parameter with a normal distribution. For the anisotropic model, it was assumed the different peer effects were independent of each other. This assumption allows for the peer effect to be individual specific, capturing potential and unobserved reference groups, given data was not available to identify reference groups. We estimated the models as outlined below following [21] and tested if potential selection bias was present using Likelihood Ration tests. Test results rejected that the peer effects should be modeled as random parameters at the 5% level of significance. The results lend support to the assumption that selection bias effects, if present, do not significantly affect regression estimates. Thus, peer effect parameters are modeled as nonrandom. Failure to model these effects as endogenous could bias results and lead to erroneous inferences [19]. It is likely then that the individual specific variables, (***X***_*i*_, ***Z***_*i*_) and their associated means, are not functions of or vary significantly by the presence of unobserved reference groups in the classroom. In this case, the pure endogenous effects modeled, if significant, will to some degree capture peer effects, but this should be tempered by limitations discussed earlier about the observed data.

Estimation of the models with anisotropic and isotropic effects is completed using a two stage estimation process to account for the endogeneity of the GPA of the students and that allows for an instrumental variables estimation approach to estimate the parameters of the spatial peer effects in the model [8,20]. GPA represents the current semester GPA for a student taking the course that semester and will be dependent on the performance in that class. It is utilized to account for a student’s performance in their other classes that semester. Thus, GPA is an endogenous variable. In addition, an instrumental variables estimation approach allows us to estimate the coefficients on the spatially lagged variables or peer effects (**W**_R_*G*_*i*_, **W**_L_*G*_*i*_, **W**_DR_*G*_*i*_, **W**_F_*G*_*i*_, **W**_DL_*G*_*i*_), by treating them as endogenous. Following [8], the student-specific independent variables (excluding GPA) and the spatial lags of the independent variables (e.g. **W**_R_***X***_*i*_, **W**_L_***X***_*i*_, **W**_DR_***X***_*i*_, **W**_F_***X***_*i*_, **W**_DL_***X***_*i*_. **W**_R_***Z***_*i*_, **W**_L_***Z***_*i*_, **W**_DR_***Z***_*i*_, **W**_F_***Z***_*i*_, **W**_DL_***Z***_*i*_) are specified as instruments to take account of the endogeneity of the spatial lags. For GPA, we include the independent variables (excluding GPA), spatially lagged GPA (i.e. **W***Z*_***i***_), and the number of hours enrolled as instruments. Given the relationship between GPA and *G*_*i*_, we felt that the spatially lagged variate would provide a better instrument as it may not be strongly correlated with individual course performance. In addition, the number of hours enrolled helps to capture some of the impact of all other classes taken that semester on cumulative GPA.

In the first stage of estimation, GPA and the spatially lagged dependent variables are regressed on the set of instruments using ordinary least squares. Using the resulting estimated parameters from the regression, instrumented regressors of the endogenous variables are then calculated to include in the second stage that models Eq (1) for both anisotropic and isotropic peer effects given by Eqs (2) and (3). Instrumental regression results for each model are provided in the supplemental appendix (S1, S2, S3, S4 and S5 Tables).

The second stage estimates the regressions given by Eq (1) for both anisotropic and isotropic peer effects given by Eqs (2) and (3). Given that the dependent variable is constrained to be between 0 and 1 (0% and 100%) with grades for individual students potentially accumulating at both endpoints, the regression given by Eq (1) is estimated as a censored or Tobit regression model following [21] with the endogenous variables replaced with their instrumented regressors. In addition, it is assumed that the intercept (*α*) is a random parameter, allowed to randomly vary across students following a normal distribution. This assumption allows for student preferences concerning teaching styles and environment, as well as differences in learning abilities to vary across students. Furthermore, it is assumed that this variation is random and the variation is unobserved by the modelers. Failure to capture this variation could result in biased and inconsistent estimates. Finally, data for both years are pooled and a dummy variable for 2010 is included in the regression to capture year specific fixed effects. The random parameter Tobit model following Eq (1) is estimated in LIMDEP 9.0 using a simulated maximum likelihood estimation procedure following [21] using 1000 Halton draws and the BFGS QuasiNewton algorithm with an increased tolerance for the norm of the gradient of 1e-8.

## Results

Estimation results for the random intercept censored tobit model of student performance for both anisotropic and isotropic effects with fit statistics are included in Tables 2 and 3 for each of the four exams (3 regular exams and 1 comprehensive final exam) and the overall course grade. The fixed effect for 2010 (“Year”) was statistically significant for the Exam 2 and Final Exam models, indicating potential differences in exams between the two years, material covered, or some other unobserved effect. Estimates of the mean and standard deviation of the random parameter intercept were significant across almost all models at a 1 percent level of significance. This indicates the that student preferences and abilities likely varied, resulting in unobserved heterogeneity that needed to be captured by the model. Finally, the results of the instrumented regressions are provided in five supplementary tables (S1, S2, S3, S4 and S5 Tables), which indicated that the instrument, hours enrolled, was a good predictor of GPA, and the spatially lagged independent variables provided good instruments for the spatially lagged dependent variables for exam and overall class performance. The sign on the hours enrolled in the instrumented regression was positive and statistically significant at the 1% level of significance. This result suggests that as students enroll in more hours, GPA increases, which may signify that students who perform well academically are up to the challenge of taking more classes in a given semester and are able to handle the additional academic load. Finally, it should be noted that effects of different explanatory variables did vary across exam and final grade outcomes, but effects were not consistent. It may be the case that students find that the first or final exam (exam 4) may be more difficult than the other exams. This may result from a student learning about a professor’s testing methodology on the first exam and the comprehensive nature of the final exam. Examination of results though, shows that the effect of different explanatory factors varied and did not necessarily increase on potentially more difficult exams. For example, the magnitude of the effect of sitting in the back of the classroom was greater for exam 2 than exam 1 or exam 4.

**Table 2:** Tobit model estimation results for student performance model with isotropic endogenous peer effects.

Variable/Effect	Exam 1	Exam 2	Exam 3	Final Exam	Overall Grade
Female	-0.019**	-0.067***	-0.048**	-0.052***	-0.044***
	(0.0089)	(0.018)	(0.023)	(0.014)	(0.015)
Algebra	-0.014*	-0.000037	-0.018	-0.0028	-0.013
	(0.0074)	(0.017)	(0.018)	(0.014)	(0.0090)
Ag Econ Major	0.000036	0.0082	0.038**	0.022	0.015*
	(0.0075)	(0.018)	(0.018)	(0.017)	(0.0090)
Non Ag Major	0.027**	0.023	0.027	0.029	0.027*
	(0.011)	(0.027)	(0.035)	(0.027)	(0.016)
Sophomore	0.035***	0.010	0.0040	0.0057	0.011
	(0.0071)	(0.016)	(0.016)	(0.015)	(0.0086)
Junior	0.034***	0.018	0.049*	0.022	0.032**
	(0.010)	(0.025)	(0.027)	(0.023)	(0.014)
Senior	0.026	-0.013	-0.015	-0.021	-0.022
	(0.017)	(0.046)	(0.046)	(0.038)	(0.027)
Middle	-0.024**	-0.024	-0.00066	0.0056	0.0066
	(0.011)	(0.030)	(0.032)	(0.025)	(0.014)
Back	-0.031***	-0.066**	-0.034	-0.055**	-0.024*
	(0.011)	(0.028)	(0.028)	(0.024)	(0.014)
Instrumented GPA	0.095***	0.20***	0.16***	0.21***	0.18***
	(0.030)	(0.057)	(0.062)	(0.033)	(0.061)
Homework 1	0.10	---	---	---	---
	(0.085)				
Homework 2	---	-0.11	---	---	---
		(0.17)			
Homework 3	---	---	0.10	---	---
			(0.17)		
Homework 4	---	---	---	-0.094	---
				(0.062)	
Overall Homework Score	---	---	---	---	-0.013
					(0.24)
Year	0.0025	-0.083***	0.022	-0.049**	-0.0071
	(0.0098)	(0.024)	(0.026)	(0.021)	(0.012)
*Isotropic Peer Effects*					
Peer Effect	0.13***	-0.047	0.12	0.077	0.054
	(0.043)	(0.10)	(0.082)	(0.070)	(0.038)
*Random Intercept*					
Mean	0.28***	0.31***	0.12*	0.25***	0.25***
	(0.039)	(0.075)	(0.072)	(0.067)	(0.039)
Standard Deviation	0.098***	0.044***	0.033***	0.020***	0.016***
	(0.0032)	(0.0067)	(0.0067)	(0.0059)	(0.0035)
σ_μ_	0.057***	0.13***	0.13***	0.11***	0.065***
	(0.0022)	(0.0041)	(0.0044)	(0.0038)	(0.0023)
*Fit Statistics*					
Log-Likelihood	263.30	187.82	201.01	259.45	444.69
AIC	-494.6	-343.6	-370.0	-486.9	-857.4
*N*	347	347	347	347	347

Note:

***, **, * indicate significance at 1%, 5%, 10% level, respectively.

Standard errors are reported in parentheses.

**Table 3:** Tobit model estimation results for student performance model with anisotropic endogenous peer effects.

Variable/Effect	Exam 1	Exam 2	Exam 3	Final Exam	Overall Grade
Female	-0.016	-0.068***	-0.055**	-0.051***	-0.040***
	(0.019)	(0.019)	(0.023)	(0.014)	(0.015)
Algebra	-0.017	-0.0016	-0.023	-0.0039	-0.013
	(0.015)	(0.017)	(0.018)	(0.015)	(0.010)
Ag Econ Major	0.0016	0.0089	0.042**	0.023	0.017
	(0.015)	(0.018)	(0.019)	(0.016)	(0.0092)
Non Ag Major	0.032	0.028	0.035	0.023	0.030*
	(0.023)	(0.028)	(0.036)	(0.028)	(0.016)
Sophomore	0.032**	0.0090	0.0022	0.00079	0.0097
	(0.015)	(0.017)	(0.016)	(0.015)	(0.0086)
Junior	0.036*	0.017	0.051*	0.019	0.031**
	(0.021)	(0.026)	(0.027)	(0.024)	(0.014)
Senior	0.018	-0.021	-0.033	-0.023	-0.022
	(0.034)	(0.046)	(0.045)	(0.038)	(0.028)
Middle	-0.029	-0.017	0.0023	0.0023	0.0071
	(0.022)	(0.031)	(0.033)	(0.026)	(0.015)
Back	-0.039*	-0.062**	-0.037	-0.055**	-0.027*
	(0.021)	(0.029)	(0.028)	(0.024)	(0.014)
Instrumented GPA	0.11*	0.20***	0.20***	0.22***	0.18***
	(0.061)	(0.058)	(0.059)	(0.036)	(0.062)
Homework 1	0.072	---	---	---	---
	(0.17)				
Homework 2	---	-0.11	---	---	---
		(0.18)			
Homework 3	---	---	0.013	---	---
			(0.16)		
Homework 4	---	---	---	-0.11	---
				(0.071)	
Overall Homework Score	---	---	---	---	0.019
					(0.25)
Year	-0.0031	-0.072***	0.017	-0.054**	-0.0077
	(0.018)	(0.025)	(0.025)	(0.022)	(0.013)
*Anisotropic Peer Effects*					
Right Side	-0.013	0.012	0.014	-0.028	0.0023
	(0.022)	(0.030)	(0.027)	(0.024)	(0.013)
Diagonal Right	-0.032	-0.034	-0.43*	0.0090	-0.018
	(0.022)	(0.028)	(0.025)	(0.021)	(0.013)
Front	0.0090	-0.019	0.0043	0.037*	0.0021
	(0.023)	(0.029)	(0.025)	(0.022)	(0.013)
Diagonal Left	0.026	-0.0041	0.026	-0.039	0.0052
	(0.021)	(0.025)	(0.023)	(0.024)	(0.012)
Left Side	-0.035	-0.018	-0.0050	0.0046	-0.0071
	(0.022)	(0.029)	(0.024)	(0.025)	(0.013)
*Random Intercept*					
Mean	0.38***	0.31***	0.19***	0.31***	0.30***
	(0.050)	(0.052)	(0.059)	(0.056)	(0.033)
Standard Deviation	0.025***	0.041***	0.034***	0.0079	0.0096***
	(0.0060)	(0.0068)	(0.0067)	(0.0059)	(0.0036)
σ_μ_	0.11***	0.13***	0.13***	0.11***	0.066***
	(0.0049)	(0.0043)	(0.0043)	(0.0038)	(0.0024)
*Fit Statistics*					
Log-Likelihood	264.81	189.22	201.93	261.99	445.21
AIC	-489.6	-338.4	-363.9	-484.0	-850.40
*N*	347	347	347	347	347

Note:

***, **, * indicate significance at 1%, 5%, 10% level, respectively.

Standard errors are reported in parentheses

### Endogenous peer effects

Endogenous peer effects are captured by the coefficient estimates on the isotropic and anisotropic spatial lags in the Tobit regression models estimated. The endogenous peer effects captured are nonparametric in nature and cannot provide a direct causal explanation of why peer effects exist in the classroom. In addition, any results discussed here should be weighed against the limitations of this study as previously discussed.

Very few of the anisotropic effects are statistically significant in Table 3. For those that are statistically significant (“diagonal right” on exam 3 and “front” on the final exam), the signs are opposite of each other. The positive and significant coefficient on exam 3 for the student sitting in front of another student, could be the result of academic dishonesty, but this would require more evidence to conclude. Future research may be able to identify this by identifying specific peer effects and isolating those specific to academic dishonesty. It is much more unclear why the result for the student in front and to the right (“diagonal right”) would be negative. This could be an example of “bad” cheating or the outcome from a student copying from another poor performing student. Again, the estimation results do not allow us to make such a conclusion. Overall, it seems that there were no significant directional peer effects in the class. While this result does not preclude the possibility of such peer effects in other classrooms (or related effects due to the presence of reference groups), it seems that there were no significant pure endogenous peer effects in this class. This is in contrast to findings by Hong and [16] who found significant endogenous peer effects, but their study was for classes in a university setting in South Korea, which may result in cultural and teaching climate that could explain the potential differences. For an instructor in this course, these findings could provide some evidence that use of a self-assigned seating arrangement by students in this situation may have limited effect at promoting positive spillovers between students, but this is in light of the fact that no reference group information was collected.

These findings are further supported by the insignificance of many of the peer effect coefficients in the isotropic models in Table 2, which were found to be relatively insignificant by [16], as well. The only significant isotropic spatial peer effect was for the first exam, which was positive and statistically significant at the 1% level. This result could indicate the potential for positive spillover effects from peers as students learn about class dynamics and teaching methods at the beginning of a course. Again, such a result cannot be explicitly concluded and requires further investigation. Future research with greater controls will need to be conducted to disentangle these effects.

### Spatial heterogeneity and proximity effects

As expected from prior literature, the area a student chooses to sit in the classroom matters. Based on results for both the isotropic and anisotropic models, students who sat in the back of the classroom on average performed worse on each exam and overall in the course. The impact on grades, as a percentage, ranged from 2.5 to 6.6 percent. That is, up to half a letter grade for this class. The results were mixed across exams. These results both concur with those found by [5,7,12,15]. Like previous research though, we cannot say whether it is because of sitting in the back students performed worse or if students who are less motivated choose to sit in the back of the class. Prior research suggests that this may due to students paying less attention, different learning orientations, lack of motivation or low classroom self-esteem [15]. It is possible that in large lecture halls students are less likely to be distracted and more motivated to stay engaged when they sit in the front of the classroom.

### Individual specific characteristics

For the other student characteristics examined, gender, choice of major, class rank and cumulative GPA had statistically significant impacts on exam and overall class performance. The coefficient on female was negative and statistically significant at the 1 to 5 percent level for all of the models, except for the model for Exam 1 with anisotropic effects. Previous studies have found mixed results for gender. While [7] found being female to be a significant predictor of higher GPAs, [3] found male students performed significantly better than female students in introductory economics courses. [2] found female students generally perform better on essay exams but male students performed better on multiple choice questions.

Being an agricultural economics major had a positive and significant effect on the exam 3 score and in the overall course grade. This may be the result of students being more motivated to take courses directly related to their major or the students had a stronger intuition regarding the concepts. In addition, non-agricultural majors performed better on exam 1 and in the overall course, indicating that other agricultural majors had a harder time performance-wise in the course.

Class rank (sophomore, junior, or senior) had mixed effects on exam scores and the overall grade relative to freshman taking the course. Interestingly, when class ranks effects were statistically significant, they were positive, indicating better performance on exams and in the overall class, which occurred more often for sophomores and juniors. The authors do not have a strong sense of why this was the case. It is possible that some of the upper classmen were non-majors as this course is generally taken by freshman majors, students may have been retaking the course, or students may have previously taken other courses such as principles of macroeconomics, which may have helped to prepare them for this course.

The instrumented GPA, a measure of preparedness and overall academic success, was positive and significant (often at the 1% level of significance) for all exams and overall class performance for both the isotropic and anisotropic models. This result is supported by prior literature, including [1,6,16]. This result could indicate that positive performance in current and past courses likely has a positive spillover effect into the present course being examined. Interestingly, performance on homework and quizzes related to the exams had no statistically significant impact on exam or overall class performance for any of the models.

## Summary

Overall, the results of this study found that directional and nondirectional pure endogenous peer effect measures did not have a statistically significant impact on performance in a large principles of agricultural economics class where students have the choice to choose where they sit, as is often the case in large lectures halls in university settings. The results may provide relief for some instructors contemplating seating arrangements for their classrooms. Such instructors could be expecting positive spillovers from significant peer effects or may be trying to avoid potential negative spillovers (e.g. from social distractions or academic dishonesty). The results suggest that spatial peer effects in the classroom may not be as significant as the spatial arrangement of the room, how courses are taught, student preparedness and organization. Additionally, from these results it indicated there was little to no nefarious activity (i.e. student dishonesty or academic cheating) potentially going on, as the methods utilized are one way to examine if spatial peer effects are leading to such activity in the classroom. On the other hand, a significant spatial effect does exist for students that choose to sit in the back of the class. Findings suggest students who sit in the back of the class will perform worse on both exams and overall in the class. This is consistent with previous studies; however, this study does not attempt to identify why students chose to sit in the back of the class room, which could be due to students’ disinterest in the subject matter, being less motived, preference for not being called on, feeling of being more at ease in the back, or simply were late getting to the classroom when seats were chosen, leaving only seats in the back row.

Many of the student or individual specific effects were found to be consistent with previous findings in the literature, including that male students on average performed better than female students in economics courses and more specifically on multiple choice exams. Furthermore, better performance in past and concurrent classes may result in a positive spillover or boost in performance in principles of economics classes. This result provides some emphasis on the importance of preparing students on how to learn, take notes, study, etc. for college.

Future research could include a survey of students asking why they chose specific seat locations and their relationship to the students sitting near them to identify reference groups and social networks that form prior and in the classroom. Additionally, the survey could include questions regarding studying habits (including whether they studied with the students sitting near them), transfer credits, housing situation, interest in taking this course, attempts to seek outside assistance, and employment during the semester. This information would help to begin to disentangle the spatial peer effects and provide much more explanatory power for this type of analysis.

## Supporting information

S1 Table Instrumental OLS regression results for the 1^st^ exam performance. (DOCX)

S2 Table Instrumental OLS regression results for the 2^nd^ exam performance. (DOCX)

S3 Table Instrumental OLS regression results for the 3^rd^ exam performance. (DOCX)

S4 Table Instrumental OLS regression results for the 4^th^ exam performance. (DOCX)

S5 Table Instrumental OLS regression results for the overall class performance. (DOCX)

S1 Text Supplementary text. (DOCX)

S1 Data Minimal data set. (XLSX)

## References

[1] Clauretie TM, Johnson EW. Factors affecting student performance in principles of economics. The Journal of Economic Education. 1975; 6(2): 132–134.

[2] Lumsden KG, Scott A. The economics student reexamined: male-female differences in comprehension. The Journal of Economic Education. 1987; 18(4): 365–375.

[3] Anderson G, Benjamin D, Fuss MA. The determinants of success in university introductory economics courses. The Journal of Economic Education. 1994; 25(2): 99–119.

[4] Ballard CL, Johnson MF. 2004. Basic math skills and performance in an introductory economics class. The Journal of Economic Education. 2004; 35(1): 3–23.

[5] Benedict ME, Hoag J. Seating location in large lectures: are seating preferences or location related to course performance? The Journal of Economic Education. 2004; 35(3): 215231.

[6] Caviglia-Harris J.L. Attendance and achievement in economics: investigating the impact of attendance policies and absentee rates on student performance. Journal of Economics and Finance Education. 2006; 4(2): 1–15.

[7] Gossard MH, Jessup E, Casavant K. Anatomy of a classroom: an exploratory analysis of elements influencing academic performance. North American Colleges and Teachers of Agriculture Journal. 2006; 50(2): 36–39.

[8] Arbia G. A Primer for Spatial Econometrics with Applications in R. 2014; New York, NY: Palgrave MacMillan.

[9] Zimmerman D. Peer effects in academic outcomes: evidence from a natural experiment. The Review of Economics and Statistics. 2003; 85(1): 9–23.

[10] Stinebrickner TR, Stinebrickner R. What can be learned about peer rffects using college roommates? Evidence from new survey data and students from disadvantaged backgrounds. CIBC Working Paper No. 2005–4. 2005; Available at: http://economics.uwo.ca/chcp/workingpapers_docs/wp2005/Stinebrickner04.pdf.

[11] Hoxby C. Peer effects in the classroom: learning from gender and race variation. NBER Working Paper No. 7867. 2000; Available at: http://www.nber.org/papers/w7867.

[12] Marshall PD, Losonczy-Marshall M. 2010. Classroom ecology: relations between seating location, performance, and attendance. Psychological Reports 2010; 107(2): 567–577. 10.2466/11.22.PR0.107.5.567-577 21117484

[13] Tagliacolloab VA, Volpatoac GL, Pereira A Jr. Association of student position in classroom and school performance. Educational Research 2010; 1(6): 198–201.

[14] Hammonds F, Mariano G. Student test grades in college: a study of possible predictors. International Journal of Teaching and Learning in Higher Education 2015; 27: 114–118.

[15] Shernoff DJ, Sannella AJ, Schorr RY, Sanchez-Wall L, Ruzek EA, Sinha S, Bressler DM. Separate worlds: the influence of seating location on student engagement, classroom experience, and performance in the large university lecture hall. Journal of Environmental Psychology 2017; 49: 55–64.

[16] Hong SC, Lee J. Who is sitting next to you? Peer effects inside the classroom. Quantitative Economics 2017; 8: 239–275.

[17] Lacroix K, Lacroix S. Does seat location matter? A review of the proximity effect is large and small classrooms. Community College Enterprise. 2017; 23: 58–69.

[18] Bergtold JS, Yeager EA, Griffin TW. Academic, demographic and spatial factors in the classroom affecting student performance in principles of agricultural economics courses. Selected paper presented at the Agricultural and Applied Economics Association Annual Meetings. 2016; Boston, MA.

[19] Manski CF. Identification of endogenous social effects: the reflection problem. Review of Economic Studies. 1993; 60: 531–542.

[20] Anselin L. Spatial Econometrics: Methods and Models. 1988; The Netherlands: Kluwer Academic Publishers.

[21] Greene WH. Econometric Analysis. 7 ed 2012; Upper Saddle River, NJ: Prentice Hall.

