# Supplementary material for: Spatial dynamics in the classroom: Does seating choice matter?
Source: PLoS One. 2019 Dec 31;14(12):e0226953. doi: 10.1371/journal.pone.0226953 (PMC6938342; doi:10.1371/journal.pone.0226953)
Supplement: S1 Table — (DOCX) [file pone.0226953.s001.docx]

S1 Table: Instrumental OLS Regression Results for the 1^st^ Exam Performance.

| Variable | GPA | Isotropic Spatially Weighted Exam Score | Exam Score to the Right | Exam Score to the Diagonal Right | Exam Score to the Front | Exam Score to the Diagonal Left | Exam Score to the Left |
| --- | --- | --- | --- | --- | --- | --- | --- |
| Intercept | -0.085 | -0.098*** | 0.0036 | 0.024 | 0.069 | 0.058 | 0.0027 |
|  | (0.32) | (0.035) | (0.048) | (0.045) | (0.049) | (0.045) | (0.046) |
| Homework | 2.53*** | -0.026 | -0.039 | -0.051 | -0.042 | 0.0021 | -0.015 |
|  | (0.25) | (0.022) | (0.039) | (0.038) | (0.039) | (0.038) | (0.038) |
| Female | 0.20*** | 0.0057 | 0.0085 | 0.0099 | -0.021* | -0.0033 | 0.0024 |
|  | (0.078) | (0.0068) | (0.012) | (0.012) | (0.012) | (0.012) | (0.012) |
| Hours Enrolled | 0.053*** | 0.0021* | 0.0040 | 0.0023 | -0.0012 | -0.0023 | 0.0031 |
|  | (0.020) | (0.0017) | (0.0032) | (0.0030) | (0.0032) | (0.0030) | (0.0031) |
| Algebra | 0.086 | -0.012 | 0.0099 | 0.0007 | 0.0089 | 0.025** | -0.028** |
|  | (0.084) | (0.0073) | (0.013) | (0.013) | (0.013) | (0.013) | (0.013) |
| Ag Econ Major | 0.067 | 0.015** | 0.012 | 0.021 | 0.023 | 0.0015 | -0.0045 |
|  | (0.091) | (0.0078) | (0.014) | (0.014) | (0.015) | (0.014) | (0.014) |
| Non Ag Major | 0.0005 | 0.010 | -0.044* | 0.0010 | 0.014 | 0.027 | 0.018 |
|  | (0.14) | (0.012) | (0.022) | (0.022) | (0.022) | (0.022) | (0.022) |
| Sophmore | 0.013 | -0.012 | -0.023* | -0.015 | -0.0033 | 0.023* | 0.0043 |
|  | (0.087) | (0.0076) | (0.014) | (0.014) | (0.014) | (0.013) | (0.014) |
| Junior | -0.15 | -0.0036 | 0.0024 | -0.027 | 0.0054 | 0.0020 | -0.029 |
|  | (0.11) | (0.0099) | (0.018) | (0.018) | (0.019) | (0.017) | (0.018) |
| Senior | 0.33** | 0.0084 | -0.0035 | -0.010 | 0.037 | 0.017 | -0.0005 |
|  | (0.16) | (0.014) | (0.026) | (0.025) | (0.026) | (0.025) | (0.025) |
| W_Homework | --- | 0.38*** | 0.045*** | 0.045*** | 0.45*** | 0.49*** | 0.46*** |
|  |  | (0.039) | (0.042) | (0.042) | (0.041) | (0.042) | (0.040) |
| W_Female | --- | -0.0084* | 0.0040 | -0.0026 | 0.0031 | 0.0018 | 0.017 |
|  |  | (0.012) | (0.014) | (0.014) | (0.014) | (0.013) | (0.013) |
| W_Hours Enrolled | --- | 0.021*** | 0.021*** | 0.023*** | 0.023*** | 0.021*** | 0.021*** |
|  |  | (0.0026) | (0.0025) | (0.0025) | (0.0025) | (0.0024) | (0.0024) |
| W_Algebra | --- | -0.024 | -0.0026 | 0.015 | -0.010 | -0.015 | -0.022 |
|  |  | (0.014) | (0.015) | (0.015) | (0.015) | (0.015) | (0.015) |
| W_Ag Econ Major | --- | 0.038*** | 0.024 | 0.025 | 0.022 | 0.026* | 0.029* |
|  |  | (0.014) | (0.016) | (0.016) | (0.016) | (0.016) | (0.015) |
| W_Non Ag Major | --- | 0.056*** | 0.044* | 0.040 | 0.070*** | 0.076*** | 0.032 |
|  |  | (0.022) | (0.024) | (0.028) | (0.026) | (0.024) | (0.028) |
| W_Sophmore | --- | 0.046*** | 0.054*** | 0.058*** | 0.047*** | 0.037*** | 0.031** |
|  |  | (0.013) | (0.015) | (0.016) | (0.015) | (0.015) | (0.015) |
| W_Junior | --- | 0.0071 | 0.042** | 0.046** | 0.020 | 0.025 | 0.021 |
|  |  | 0.021 | (0.020) | (0.020) | (.021) | (0.021) | (0.020) |
| W_Senior | --- | 0.058*** | 0.032 | 0.045* | 0.026 | 0.023 | 0.051* |
|  |  | 0.021 | (0.029) | (0.028) | (0.029) | (0.027) | (0.028) |
| W_GPA | -0.006 | --- | --- | --- | --- | --- | --- |
|  | (0.070) |  |  |  |  |  |  |
| R^2^ | 0.32 | 0.64 | 0.87 | 0.90 | 0.86 | 0.89 | 0.87 |
| *N* | 347 | 347 | 347 | 347 | 347 | 347 | 347 |

Note: “W_” indicates a spatially weighted variable. ***, **, * indicate significance at 1%, 5%, 10% level, respectively. Standard errors are reported in parentheses.
