# Supplementary material for: Spatial dynamics in the classroom: Does seating choice matter?
Source: PLoS One. 2019 Dec 31;14(12):e0226953. doi: 10.1371/journal.pone.0226953 (PMC6938342; doi:10.1371/journal.pone.0226953)
Supplement: S2 Table — (DOCX) [file pone.0226953.s002.docx]

S2 Table: Instrumental OLS Regression Results for the 2^nd^ Exam Performance.

| Variable | GPA | Isotropic Spatially Weighted Exam Score | Exam Score to the Right | Exam Score to the Diagonal Right | Exam Score to the Front | Exam Score to the Diagonal Left | Exam Score to the Left |
| --- | --- | --- | --- | --- | --- | --- | --- |
| Intercept | -0.31 | -0.009 | -0.11** | -0.0085 | -0.028 | 0.035 | 0.022 |
|  | (0.32) | (0.043) | (0.055) | (0.047) | (0.053) | (0.053) | (0.055) |
| Homework | 2.71*** | 0.0019 | 0.0053 | 0.0092 | -0.068* | -0.042 | -0.041 |
|  | (0.23) | (0.025) | (0.044) | (0.039) | (0.042) | (0.043) | (0.044) |
| Female | 0.21*** | 0.023*** | 0.024* | 0.013 | 0.023* | 0.085 | 0.016 |
|  | (0.075) | (0.0080) | (0.014) | (0.012) | (0.013) | (0.014) | (0.014) |
| Hours Enrolled | 0.066*** | 0.0038* | 0.0083** | 0.0012 | 0.0039 | 0.0002 | 0.0007 |
|  | (0.019) | (0.0020) | (0.0035) | (0.0031) | (0.0034) | (0.034) | (0.0035) |
| Algebra | 0.047 | 0.0018 | 0.0161 | 0.0063 | -0.0067 | -0.0008 | -0.0035 |
|  | (0.081) | (0.0086) | (0.0153) | (0.014) | (0.015) | (0.015) | (0.015) |
| Ag Econ Major | 0.070 | 0.024*** | -0.0036 | 0.0024 | 0.040*** | 0.023 | 0.0086 |
|  | (0.088) | (0.0092) | (0.016) | (0.015) | (0.016) | (0.016) | (0.017) |
| Non Ag Major | 0.10 | 0.024* | -0.023 | 0.0031 | 0.027 | 0.027 | 0.012 |
|  | (0.14) | (0.014) | (0.026) | (0.023) | (0.024) | (0.025) | (0.026) |
| Sophmore | 0.045 | -0.014* | -0.032** | -0.023** | 0.031** | -0.028* | 0.0005 |
|  | (0.084) | (0.0089) | (0.016) | (0.014) | (0.015) | (0.016) | (0.016) |
| Junior | -0.054 | -0.0013 | -0.0078 | -0.035* | -0.012 | 0.0023 | 0.020 |
|  | (0.11) | (0.012) | (0.021) | (0.019) | (0.020) | (0.020) | (0.021) |
| Senior | 0.26* | 0.0060 | -0.0008 | -0.035 | 0.034 | 0.024 | -0.0058 |
|  | (0.16) | (0.17) | (0.030) | (0.027) | (0.028) | (0.029) | (0.030) |
| W_Homework | --- | 0.27*** | 0.49*** | 0.51*** | 0.42*** | 0.46*** | 0.44*** |
|  |  | (0.041) | (0.045) | (0.046) | (0.044) | (0.047) | (0.045) |
| W_Female | --- | -0.011 | -0.030* | -0.023 | -0.0089 | -0.0026 | -0.0047 |
|  |  | (0.014) | (0.016) | (0.015) | (0.015) | (0.015) | (0.016) |
| W_Hours Enrolled | --- | 0.028*** | 0.020*** | 0.020*** | 0.024*** | 0.022*** | 0.023*** |
|  |  | (0.0028) | (0.0026) | (0.0026) | (0.0025) | (0.0026) | (0.0026) |
| W_Algebra | --- | 0.014 | -0.0008 | 0.0082 | 0.025 | 0.024 | 0.013 |
|  |  | (0.016) | (0.017) | (0.016) | (0.016) | (0.017) | (0.017) |
| W_Ag Econ Major | --- | 0.070*** | 0.044*** | 0.030* | 0.042*** | 0.038** | 0.043*** |
|  |  | (0.017) | (0.018) | (0.017) | (0.017) | (0.018) | (0.018) |
| W_Non Ag Major | --- | 0.036 | 0.044 | 0.041 | 0.076*** | 0.073*** | 0.029 |
|  |  | (0.026) | 0.028 | (0.030) | (0.028) | (0.028) | (0.033) |
| W_Sophmore | --- | 0.052*** | 0.050*** | 0.040*** | 0.032** | 0.018 | 0.022 |
|  |  | (0.015) | (0.017) | (0.016) | (0.016) | (0.017) | (0.018) |
| W_Junior | --- | 0.024 | 0.035 | 0.038* | 0.049** | 0.017 | 0.016 |
|  |  | (0.024) | (0.023) | (0.021) | (0.022) | (0.024) | (0.023) |
| W_Senior | --- | 0.050** | 0.038 | 0.039 | 0.011 | 0.027 | 0.049 |
|  |  | (0.025) | (0.033) | (0.029) | (0.031) | (0.032) | (0.033) |
| W_GPA | 0.0044 | --- | --- | --- | --- | --- | --- |
|  | (0.068) |  |  |  |  |  |  |
| R^2^ | 0.37 | 0.54 | 0.83 | 0.88 | 0.84 | 0.85 | 0.83 |
| *N* | 347 | 347 | 347 | 347 | 347 | 347 | 347 |

Note: “W_” indicates a spatially weighted variable. ***, **, * indicate significance at 1%, 5%, 10% level, respectively. Standard errors are reported in parentheses.
