# Supplementary material for: Spatial dynamics in the classroom: Does seating choice matter?
Source: PLoS One. 2019 Dec 31;14(12):e0226953. doi: 10.1371/journal.pone.0226953 (PMC6938342; doi:10.1371/journal.pone.0226953)
Supplement: S3 Table — (DOCX) [file pone.0226953.s003.docx]

S3 Table: Instrumental OLS Regression Results for the 3^rd^ Exam Performance.

| Variable | GPA | Isotropic Spatially Weighted Exam Score | Exam Score to the Right | Exam Score to the Diagonal Right | Exam Score to the Front | Exam Score to the Diagonal Left | Exam Score to the Left |
| --- | --- | --- | --- | --- | --- | --- | --- |
| Intercept | -0.093 | 0.062 | -0.057 | -0.027 | -0.033 | 0.090* | 0.0032 |
|  | (0.030) | (0.041) | (0.051) | (0.048) | (0.053) | (0.050) | (0.052) |
| Homework | 2.52*** | 0.0097 | 0.022 | -0.024 | -0.028 | -0.035 | 0.015 |
|  | (0.20) | (0.021) | (0.037) | (0.036) | (0.037) | (0.037) | (0.037) |
| Female | 0.30*** | 0.015* | -0.013 | 0.0084 | 0.0080 | 0.018 | 0.0056 |
|  | (0.072) | (0.0079) | (0.014) | (0.013) | (0.014) | (0.013) | (0.014) |
| Hours Enrolled | 0.054*** | 0.0031 | 0.0068* | 0.0042 | 0.0047 | -0.0040 | 0.000 |
|  | (0.018) | (0.0020) | (0.0034) | (0.0033) | (0.0035) | (0.0034) | (0.0035) |
| Algebra | 0.065 | -0.0085 | -0.011 | -0.013 | -0.016 | 0.026* | -0.0060 |
|  | (0.079) | (0.0085) | (0.015) | (0.014) | (0.015) | (0.015) | (0.015) |
| Ag Econ Major | 0.0057 | 0.0025 | -0.034** | 0.012 | 0.030* | -0.010 | 0.0023 |
|  | (0.086) | (0.0093) | (0.016) | (0.016) | (0.016) | (0.016) | (0.016) |
| Non Ag Major | 0.0034 | -0.0013 | -0.042* | 0.0056 | 0.0039 | -0.020 | 0.0013 |
|  | (0.13) | (0.014) | (0.025) | (0.024) | (0.025) | (0.025) | (0.025) |
| Sophmore | 0.0028 | -0.013 | -0.027* | -0.0020 | -0.0069 | -0.016 | 0.0001 |
|  | (0.082) | (0.0089) | (0.015) | (0.015) | (0.016) | (0.015) | (0.015) |
| Junior | -0.081 | -0.013 | -0.014 | -0.029 | -0.0037 | -0.011 | 0.0057 |
|  | (0.011) | (0.012) | (0.020) | (0.020) | (0.021) | (0.020) | (0.020) |
| Senior | 0.44*** | 0.020 | 0.026 | 0.018 | 0.0095 | -0.0045 | 0.018 |
|  | (0.15) | (0.017) | (0.029) | (0.028) | (0.029) | (0.028) | (0.029) |
| W_Homework | --- | 0.49*** | 0.53*** | 0.53*** | 0.051*** | 0.56*** | 0.47*** |
|  |  | (0.040) | (0.038) | (0.042) | (0.042) | (0.043) | (0.041) |
| W_Female | --- | -0.013 | 0.0074 | 0.0084 | 0.023 | 0.027 | 0.024 |
|  |  | (0.014) | (0.015) | (0.015) | (0.015) | (0.015) | (0.015) |
| W_Hours Enrolled | --- | 0.020*** | 0.022*** | 0.023*** | 0.023*** | 0.020*** | 0.025*** |
|  |  | (0.0029) | (0.0022) | (0.0025) | (0.0025) | (0.0025) | (0.0024) |
| W_Algebra | --- | -0.047*** | -0.018 | -0.0085 | -0.0046 | -0.0084 | -0.013 |
|  |  | (0.016) | (0.016) | (0.017) | (0.017) | (0.017) | (0.017) |
| W_Ag Econ Major | --- | 0.038** | 0.060*** | 0.046*** | 0.046*** | 0.039** | 0.053*** |
|  |  | (0.017) | (0.018) | (0.018) | (0.018) | (0.018) | (0.018) |
| W_Non Ag Major | --- | -0.019 | 0.021 | 0.022 | 0.031 | 0.045* | 0.067** |
|  |  | (0.026) | (0.027) | (0.031) | (0.029) | (0.028) | (0.032) |
| W_Sophmore | --- | 0.014 | 0.026 | 0.035 | 0.024 | 0.0037 | 0.0064 |
|  |  | (0.015) | (0.017) | (0.017) | (0.017) | (0.017) | (0.017) |
| W_Junior | --- | 0.038* | 0.050** | 0.066*** | 0.041* | 0.055*** | 0.049** |
|  |  | (0.023) | (0.022) | (0.022) | (0.023) | (0.023) | (0.022) |
| W_Senior | --- | 0.092*** | 0.037 | 0.062** | 0.057* | 0.062** | 0.059* |
|  |  | (0.024) | (0.031) | (0.031) | (0.032) | (0.031) | (0.032) |
| W_GPA | 0.047 | --- | --- | --- | --- | --- | --- |
|  | (0.065) |  |  |  |  |  |  |
| R^2^ |  | 0.63 | 0.86 | 0.89 | 0.85 | 0.87 | 0.86 |
| *N* | 347 | 347 | 347 | 347 | 347 | 347 | 347 |

Note: “W_” indicates a spatially weighted variable. ***, **, * indicate significance at 1%, 5%, 10% level, respectively. Standard errors are reported in parentheses.
