# Supplementary material for: Spatial dynamics in the classroom: Does seating choice matter?
Source: PLoS One. 2019 Dec 31;14(12):e0226953. doi: 10.1371/journal.pone.0226953 (PMC6938342; doi:10.1371/journal.pone.0226953)
Supplement: S4 Table — (DOCX) [file pone.0226953.s004.docx]

S4 Table: Instrumental OLS Regression Results for the 4^th^ Exam Performance.

| Variable | GPA | Isotropic Spatially Weighted Exam Score | Exam Score to the Right | Exam Score to the Diagonal Right | Exam Score to the Front | Exam Score to the Diagonal Left | Exam Score to the Left |
| --- | --- | --- | --- | --- | --- | --- | --- |
| Intercept | 0.089 | 0.044 | -0.079* | -0.0087 | 0.029 | 0.040 | -0.0014 |
|  | (0.34) | (0.035) | (0.047) | (0.043) | (0.045) | (0.045) | (0.046) |
| Homework | 1.53*** | 0.031* | 0.013 | 0.0046 | 0.025 | -0.015 | 0.046* |
|  | (0.19) | (0.016) | (0.029) | (0.027) | (0.028) | (0.028) | (0.029) |
| Female | 0.24*** | 0.0063 | 0.0051 | 0.013 | -0.012 | 0.014 | -0.0095 |
|  | (0.081) | (0.0068) | (0.012) | (0.012) | (0.012) | (0.012) | (0.012) |
| Hours Enrolled | 0.077*** | 0.0023 | 0.0074*** | 0.0005 | -0.0024 | -0.0003 | -0.0014 |
|  | (0.020) | (0.0017) | (0.0031) | (0.0029) | (0.0030) | (0.0030) | (0.0031) |
| Algebra | 0.057 | 0.0001 | -0.017 | 0.014 | 0.013 | 0.0029 | 0.0035 |
|  | (0.088) | (0.0073) | (0.014) | (0.013) | (0.013) | (0.013) | (0.013) |
| Ag Econ Major | 0.063 | 0.022*** | 0.0003 | 0.013 | 0.018 | 0.012 | 0.013 |
|  | (0.096) | (0.0079) | (0.015) | (0.014) | (0.014) | (0.014) | (0.014) |
| Non Ag Major | 0.033 | 0.0064 | -0.012 | -0.0070 | 0.019 | -0.019 | 0.0023 |
|  | (0.15) | (0.012) | (0.023) | (0.021) | (0.022) | (0.022) | (0.022) |
| Sophmore | 0.13 | -0.0085 | -0.018 | 0.0030 | 0.0049 | -0.026* | 0.0067 |
|  | (0.091) | (0.0076) | (0.014) | (0.013) | (0.013) | (0.014) | (0.014) |
| Junior | -0.054 | -0.0016 | 0.0038 | -0.0044 | 0.0026 | -0.025 | 0.013 |
|  | (0.12) | (0.0099) | (0.018) | (0.017) | (0.018) | (0.018) | (0.018) |
| Senior | 0.41*** | 0.011 | 0.013 | 0.0098 | 0.0047 | -0.014 | -0.0027 |
|  | (0.17) | (0.014) | (0.026) | (0.025) | (0.025) | (0.025) | (0.026) |
| W_Homework | --- | 0.24*** | 0.28*** | 0.25*** | 0.24*** | 0.32*** | 0.26*** |
|  |  | (0.030) | (0.031) | (0.031) | (0.029) | (0.032) | (0.031) |
| W_Female | --- | 0.0082 | 0.011 | 0.012 | 0.022 | 0.014 | 0.024* |
|  |  | (0.012) | (0.014) | (0.014) | (0.013) | (0.014) | (0.014) |
| W_Hours Enrolled | --- | 0.030*** | 0.033*** | 0.036*** | 0.035*** | 0.031*** | 0.035*** |
|  |  | (0.0024) | (0.0021) | (0.0021) | (0.0020) | (0.0021) | (0.0021) |
| W_Algebra | --- | 0.033 | 0.0070 | 0.0095 | 0.0077 | 0.012 | -0.0095 |
|  |  | (0.014) | (0.015) | (0.015) | (0.014) | (0.015) | (0.015) |
| W_Ag Econ Major | --- | 0.096*** | 0.070*** | 0.067*** | 0.060*** | 0.075*** | 0.065*** |
|  |  | (0.015) | (0.016) | (0.016) | (0.015) | (0.016) | (0.016) |
| W_Non Ag Major | --- | 0.043* | 0.042* | 0.063** | 0.072*** | 0.075*** | 0.052* |
|  |  | (0.022) | (0.025) | (0.028) | (0.025) | (0.025) | (0.028) |
| W_Sophmore | --- | 0.050*** | 0.057*** | 0.059*** | 0.055*** | 0.035*** | 0.035** |
|  |  | (0.013) | (0.015) | (0.015) | (0.014) | (0.015) | (0.015) |
| W_Junior | --- | 0.030 | 0.032 | 0.030 | 0.044** | 0.033 | 0.031 |
|  |  | (0.020) | (0.020) | (0.020) | (0.020) | (0.021) | (0.020) |
| W_Senior | --- | 0.10*** | 0.069*** | 0.062** | 0.048* | 0.055* | 0.071*** |
|  |  | (0.021) | (0.029) | (0.027) | (0.028) | (0.028) | (0.028) |
| W_GPA | 0.058 | --- | --- | --- | --- | --- | --- |
|  | (0.073) |  |  |  |  |  |  |
| R^2^ | 0.26 | 0.66 | 0.88 | 0.91 | 0.89 | 0.90 | 0.88 |
| *N* | 347 | 347 | 347 | 347 | 347 | 347 | 347 |

Note: “W_” indicates a spatially weighted variable. ***, **, * indicate significance at 1%, 5%, 10% level, respectively. Standard errors are reported in parentheses.
