# Supplementary material for: Spatial dynamics in the classroom: Does seating choice matter?
Source: PLoS One. 2019 Dec 31;14(12):e0226953. doi: 10.1371/journal.pone.0226953 (PMC6938342; doi:10.1371/journal.pone.0226953)
Supplement: S5 Table — (DOCX) [file pone.0226953.s005.docx]

S5 Table: Instrumental OLS Regression Results for the Overall Class Performance.

| Variable | GPA | Isotropic Spatially Weighted Exam Score | Exam Score to the Right | Exam Score to the Diagonal Right | Exam Score to the Front | Exam Score to the Diagonal Left | Exam Score to the Left |
| --- | --- | --- | --- | --- | --- | --- | --- |
| Intercept | -0.66 | 0.033 | -0.044 | -0.0087 | 0.0045 | 0.034 | 0.014 |
|  | (0.29) | (0.021) | (0.029) | (0.026) | (0.029) | (0.027) | (0.028) |
| Homework | 3.84*** | 0.018 | 0.016 | -0.0045 | 0.0091 | -0.018 | 0.0092 |
|  | (0.25) | (0.015) | (0.026) | (0.024) | (0.026) | (0.025) | (0.026) |
| Female | 0.21** | 0.0087** | 0.0033 | 0.0097 | 0.0024 | 0.0043 | 0.0050 |
|  | (0.068) | (0.0041) | (0.0072) | (0.0066) | (0.0071) | (0.0069) | (0.0071) |
| Hours Enrolled | 0.030* | 0.0009 | 0.0037** | 0.0016 | -0.0007 | -0.0006 | -0.0008 |
|  | (0.017) | (0.0011) | (0.0019) | (0.0017) | (0.0019) | (0.0018) | (0.0019) |
| Algebra | 0.096 | -0.0044 | -0.0045 | 0.0044 | -0.0022 | 0.0001 | -0.0088 |
|  | (0.074) | (0.0044) | (0.0079) | (0.0073) | (0.0078) | (0.0075) | (0.0077) |
| Ag Econ Major | -0.0079 | 0.0078* | -0.0058 | 0.0064 | 0.020** | 0.0046 | -0.0001 |
|  | (0.080) | (0.0047) | (0.0085) | (0.0079) | (0.0084) | (0.0081) | (0.0084) |
| Non Ag Major | -0.041 | 0.0018 | -0.016 | -0.011 | 0.012 | 0.0002 | 0.0007 |
|  | (0.12) | (0.0074) | (0.013) | (0.012) | (0.013) | (0.013) | (0.013) |
| Sophmore | 0.032 | -0.0091** | -0.014* | -0.014 | 0.0033 | -0.019*** | 0.0049 |
|  | (0.076) | (0.0046) | (0.0081) | (0.0076) | (0.0081) | (0.0078) | (0.0080) |
| Junior | -0.11 | -0.0067 | -0.0005 | -0.025*** | -0.0011 | -0.0075 | -0.0010 |
|  | (0.10) | (0.0059) | (0.011) | (0.01) | (0.011) | (0.010) | (0.011) |
| Senior | 0.32** | 0.0041 | 0.0072 | -0.0065 | 0.0093 | 0.0060 | -0.0035 |
|  | (0.14) | (0.0086) | (0.015) | (0.014) | (0.015) | (0.014 | (0.015) |
| W_Homework | --- | 0.71*** | 0.73*** | 0.77*** | 0.72*** | 0.78*** | 0.72*** |
|  |  | (0.027) | (0.026) | (0.028) | (0.027) | (0.027) | (0.027) |
| W_Female | --- | -0.0028 | -0.0015 | -0.000 | 0.0047 | -0.003 | 0.0077 |
|  |  | (0.0073) | (0.0080) | (0.0078) | (0.0078) | (0.0078) | (0.0079) |
| W_Hours Enrolled | --- | 0.011*** | 0.012*** | 0.011*** | 0.013*** | 0.010*** | 0.013*** |
|  |  | (0.0016) | (0.0015) | (0.0016) | (0.0016) | (0.0015) | (0.0015) |
| W_Algebra | --- | -0.0083 | -0.0023 | 0.0032 | 0.0065 | 0.0080 | 0.0012 |
|  |  | (0.0081) | (0.0087) | (0.0085) | (0.0086) | (0.0087) | (0.0088) |
| W_Ag Econ Major | --- | 0.025*** | 0.031*** | 0.018** | 0.020** | 0.020** | 0.025*** |
|  |  | (0.0089) | (0.0095) | (0.0091) | (0.0093) | (0.0093) | (0.0092) |
| W_Non Ag Major | --- | 0.0064 | 0.019 | 0.019 | 0.030* | 0.033*** | 0.024 |
|  |  | (0.013) | (0.014) | (0.016) | (0.015) | (0.014) | (0.017) |
| W_Sophmore | --- | 0.034*** | 0.030*** | 0.030*** | 0.027*** | 0.013 | 0.013 |
|  |  | (0.0079) | (0.0090) | (0.0087) | (0.0088) | (0.0086) | (0.0089) |
| W_Junior | --- | 0.0087 | 0.024** | 0.027*** | 0.027** | 0.014 | 0.020* |
|  |  | (0.012) | (0.012) | (0.011) | (0.012) | (0.012) | (0.012) |
| W_Senior | --- | 0.032*** | 0.028* | 0.031 | 0.026 | 0.025 | 0.035** |
|  |  | (0.013) | (0.017) | (0.016) | (0.017) | (0.016) | (0.017) |
| W_GPA | -0.027 | --- | --- | --- | --- | --- | --- |
|  | (0.061) |  |  |  |  |  |  |
| R^2^ | 0.48 | 0.87 | 0.96 | 0.97 | 0.96 | 0.97 | 0.96 |
| *N* | 347 | 347 | 347 | 347 | 347 | 347 | 347 |

Note: “W_” indicates a spatially weighted variable. ***, **, * indicate significance at 1%, 5%, 10% level, respectively. Standard errors are reported in parentheses.
