# Supplementary material for: Spatial dynamics in the classroom: Does seating choice matter?
Source: PLoS One. 2019 Dec 31;14(12):e0226953. doi: 10.1371/journal.pone.0226953 (PMC6938342; doi:10.1371/journal.pone.0226953)
Supplement: S1 Text — (DOCX) [file pone.0226953.s006.docx]

**S1 Instrumental Regression Results**

Results from the instrumental variable regressions are provided below for reference. All regressions were conducted using ordinary least squares. For instrumental regressions examining exam scores of neighbors, OLS results were compared with Tobit models. No differences in coefficient magnitudes, significance or sign were found when using the instrumented variables from these regressions in the performance models. Spatial lags used in examination of anisotropic peer effects matched the spatial weighting used for the associated dependent variable. The instrumental regressions for GPA and isotropic peer effects used the more general weight matrix that include all neighbors to the right, diagonal front, front, diagonal left, and left.
